# Disease severity enhancement by an esterase from non-phytopathogenic yeast *Pseudozyma antarctica* and its potential as adjuvant for biocontrol agents

**DOI:** 10.1038/s41598-018-34705-z

**Published:** 2018-11-07

**Authors:** Hirokazu Ueda, Daisuke Kurose, Soichi Kugimiya, Ichiro Mitsuhara, Shigenobu Yoshida, Jun Tabata, Ken Suzuki, Hiroko Kitamoto

**Affiliations:** 1 National Agriculture and Food Research Organization (NARO), Kan-nondai, Tsukuba, Ibaraki Japan; 2 Present Address: CABI Europe-UK, Bakeham Lane, Egham, Surrey TW20 9TY UK

## Abstract

The phylloplane yeast *Pseudozyma antarctica* secretes an esterase, named PaE, and xylanase when cultivated with xylose. We previously observed that the lipophilic layer of Micro-Tom tomato leaves became thinner after the culture filtrate treatment. The leaves developed reduced water-holding ability and became wilted. In this study, the purified enzymes were spotted on Micro-Tom leaves. PaE, but not xylanase, thinned the lipophilic layer of leaves and decreased leaf resistance to the phytopathogenic fungus *Botrytis cinerea*. Disease severity increased significantly in detached leaves and potted plants treated with the culture filtrate and *B*. *cinerea* spores compared with those treated with inactivated enzyme and *B*. *cinerea* alone. Spore germination ratios, numbers of penetrating fungal hyphae in the leaves, and fungal DNA contents also increased significantly on the detached leaves. Japanese knotweed (*Fallopia japonica*), a serious invasive alien weed in Europe and North America, also became susceptible to infection by the rust pathogen *Puccinia polygoni-amphibii* var. *tovariae* following the culture filtrate treatment. The culture filtrate treatment increased disease development in plants induced by both phytopathogenic fungi. Our results suggest that *P*. *antarctica* culture filtrate could be used as an adjuvant for sustainable biological weed control using phytopathogenic fungi.

## Introduction

Plants are exposed to various environmental stresses including infection by pathogenic microorganisms. The lipophilic layer covering the surface of plants functions primarily as a physical barrier to infection^1^. The cuticle is composed of a top wax coating and a lower lipophilic polymer layer (crosswise esterified hydroxy fatty acids) that blends with the cell wall substances^2^. Because the cuticle layers also prevent the passive diffusion of nutrients and water vapor from the plant interior onto the surface, it is thought plant-associated microorganisms have limited access to nutrients^3^. Various plant-associated yeasts can secrete a variety of hydrolytic enzymes. These enzymes have been suggested to enhance the capacity of the phylloplane yeasts to extract nutrients from plants, however, little evidence of this phenomenon has been reported.

The basidiomycete yeasts *Pseudozyma* often isolated from healthy leaf surfaces are recognized as non-pathogenic residents. *Pseudozyma antarctica* secretes a 20.4-kDa esterase named PaE, which shares 61% identity with a cutinase-like enzyme from the yeast *Cryptococcus* sp. strain S-2 (AB102945)^4^. These two enzymes have wide spectra of activities with various lengths of fatty acid esters and aliphatic polyester films that are used as biodegradable plastics (BPs)^5,6^. The enzymes share roughly 19% similarity with the cutinases of filamentous fungi, which have catalytic activity mainly on short-chain fatty acids and lower specificity for BPs^7,8^. However, the biological function of PaE on leaves has not been elucidated.

*Pseudozyma antarctica* produced large amounts of PaE when cultured with xylose^9^. The culture filtrate (crude enzyme solution) also contained relatively smaller amount of two esterases (lipases A and B) and a high concentration of endo-β-xylanase^10,11^. We treated leaves of tomato and *Arabidopsis thaliana* with the crude enzyme solution and observed that the leaves became dehydrated readily. The lipophilic cuticle layer of the treated leaves was thinner, and free fatty acids with 16- and 18-carbon chains were eluted in the reaction solution^10^. Of the three esterases, PaE showed 99.7% degradation activity toward a *para*-nitrophenyl palmitate (*p*NP-C16) film in *in vitro*. Since C-16 and C-18 fatty acids are major components of the cuticle layers of tomato and *Arabidopsis*^12,13^, PaE may have a potential role in the extraction of fatty acids from plant surfaces. *P*. *antarctica* then can assimilate them as carbon source.

On the other hand, there is a continuing need for novel weed control methods to reduce chemical herbicide use. Some phytopathogenic microorganisms have been used in classical biological control strategies, for example, as sustainable control agents of invasive alien weeds. Biocontrol agents must possess highly specific virulence to suppress target weeds while reducing treatment costs^14^. Rust fungi are biotrophic and obligate plant pathogens that are being studied as promising classical biocontrol agent. The rust fungus *Puccina polygoni-amphibii* var. *tovariae* (*Puccinia*) was selected as a potential biological control agent for *Fallopia japonica* (Japanese knotweed)^15^, which is regarded as a serious problem weed that reduces native species diversity in Europe and North America. However, because rust fungi sporulate only on the targeted plant, it is difficult to prepare large amounts of rust spore inoculum^16–19^. Increased infection efficiency reduces the required amount of spores for inoculation.

Most fungal pathogens penetrate their hosts through natural openings such as stomata or wounds, while some fungi invade by direct penetration through the plant surface. After being placed on the surfaces of leaves, spores produced cutinase in the development of disease, implying that cutinase has an important role in the penetration of the cuticle layer^20,21^. From these reports, we considered that such leaves with damaged cuticles after the enzyme treatment would be prone to infection, and enhance the effectiveness of biocontrol agents. Cutinase, one of the candidate enzymes that had been developed as adjuvant to increase the effects of herbicides^22^ might also function as an adjuvant to enhance infection of rust spores into weed. However, no cutinase that could be applied with chemical herbicides is available commercially. Recently, PaE productivity was enhanced in a genetically modified *P*. *antarctica* strain^23^, which has been proposed a candidate adjuvant for biocontrol agents. A simple affinity purification method of cutinase-like enzymes was developed based on its BPs adsorption and degradation activities^24^, which has made it possible to investigate the effect of high concentrations of purified PaE on leaves, as well as adjuvant effect on infection by biocontrol agents.

## Results

### Effect of purified enzyme on detached Micro-Tom leaves

**Figure 1:**
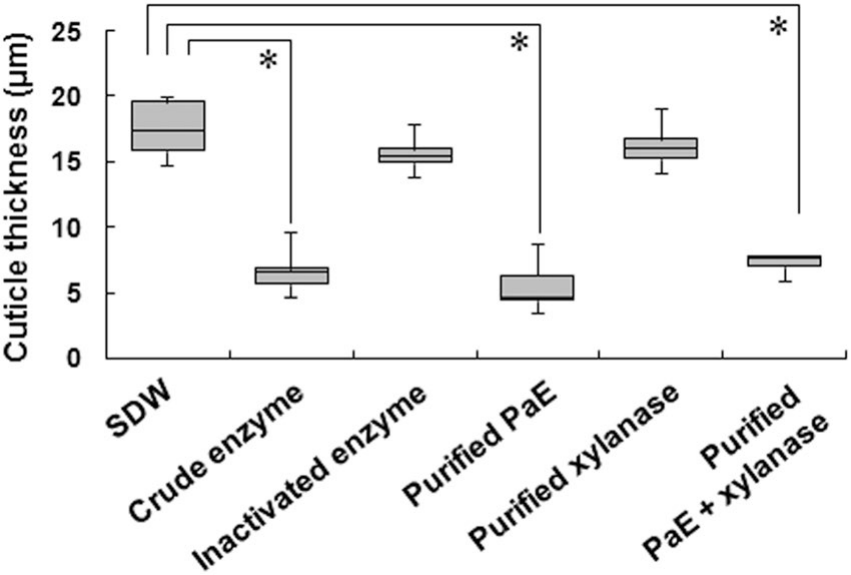
Thickness of cuticle layer of Micro-Tom leaves treated with dH_2_O, crude enzyme solution, inactivated crude enzyme solution, purified PaE, purified xylanase and mixture of purified PaE and xylanase. After treatment of each solution, cuticle was stained with Sudan III and was measured the thickness of red-stained layer under microscopy. Box plot explanation: upper horizontal line of box, 75th percentile; lower horizontal line of box, 25th percentile; horizontal bar within box, median; upper and lower lines outside the boxes, minimum and maximum values (error bars). Experiments were repeated independently (n = 6). The data analyzed by Dunnett’s test; Asterisk indicates that the data compared were significantly different (*P* < 0.01).

Previous studies have shown that the lipophilic layer covering the aerial epidermis of leaves became thinner following crude enzyme treatment^7^. PaE and xylanase^11,23^, the major protein components of the crude enzyme solution, were isolated (Figure S1). Droplets of the each purified enzyme solution and control samples were spotted on the detached Micro-Tom leaves separately. To clarify the effect of enzyme treatment on the lipophilic layer of leaves, the thickness of the lipophilic layer was measured on a cross-sectional view of leaf surfaces under a light microscope after staining the lipophilic layer with Sudan III (Figure S2). The lipophilic layer was found to have been significantly thinned by treatment of either enzyme solution that contained active PaE (crude enzyme solution, purified PaE, and enzyme mixture of purified PaE and xylanase). In contrast, the lipophilic layer of samples treated separately with inactivated enzyme solution, and purified xylanase looks the same as that of samples treated with sterilized distilled water (SDW) (control) (Fig. 1). These results indicated that PaE in crude enzyme solution but not xylanase has significant activity for thinning the lipophilic layer of plant surface. Based on SDS-PAGE analysis, the crude enzyme solution contains relatively smaller amount of two esterases (lipases A and B) compared to PaE and xylanase^10^. The effects of lipase A and B droplet treatments on leaves were examined using commercial enzymes at the same protein concentration as that of PaE. As a result, leaf thicknesses after treatment with lipase A (16.1 µm, SE ± 0.7) and B (15.0 µm, SE ± 0.8) were equivalent to those after water treatment, which was used as a control (17.3 µm, SE ± 1.0). This result suggests that the enzyme responsible for the leaf thinning effect of crude enzyme solution is a PaE.

### Susceptibility to pathogens of enzyme-treated detached Micro-Tom leaves

**Figure 2:**
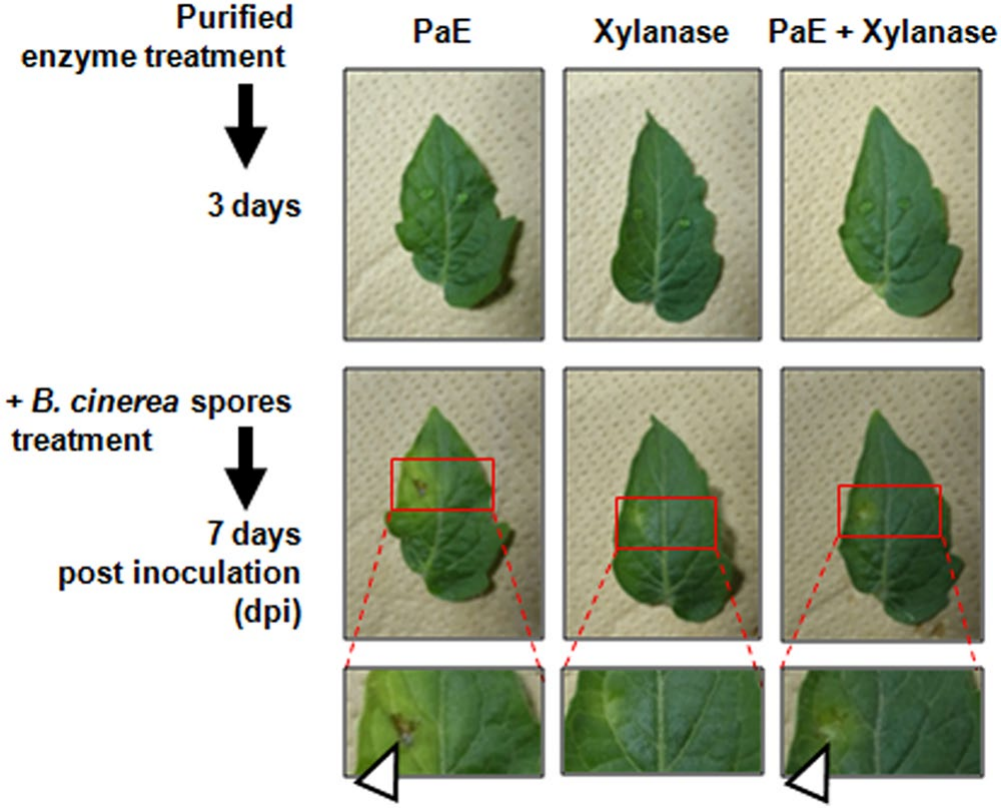
Disease development of *B*. *cinerea* on detached Micro-Tom leaves following treatment with purified enzyme solutions. The enzyme solution (purified PaE, purified xylanase, or mixture of purified PaE and xylanase) and buffer (control) were spotted on the left and right sides of the leaves, respectively. After 3 days, these leaves were inoculated with *B*. *cinerea* spores. Representative images of leaves 7 days after *B*. *cinerea* inoculation (Middle). Enlarged images of the enzyme-treated areas. Open arrow indicates a disease symptom (Bottom). Data were reproducible (n = 6).

Considering that the cuticle serves as a physical barrier against pathogen invasion^25,26^, treatment with the crude enzyme solution was expected to influence plant susceptibility to pathogens. To confirm this expectation, spore suspension of a gray mold (*B*. *cinerea*), which is one of the serious pathogens of tomatoes, were inoculated over the enzyme-spotted area on leaves (Fig. 2). On the leaves pre-treated with PaE containing solution (PaE only, and PaE + xylanase), disease symptoms (black spots) were developed within 7 days after treatment with *B*. *cinerea* spores. In contrast, no symptoms were observed on the leaves pre-treated with xylanase only.

The purified PaE and crude enzyme solution showed similar lipolytic layer-thinning effect and enhance the pathogen infectivity of treated leaves. These results indicated that main cause of the disease in the crude enzyme solution is the PaE. Therefore, we used the crude enzyme solution in subsequent large scale study.

### Disease severity in crude enzyme-treated Micro-Tom detached leaves following inoculation with *B*. *cinerea* spores

**Figure 3:**
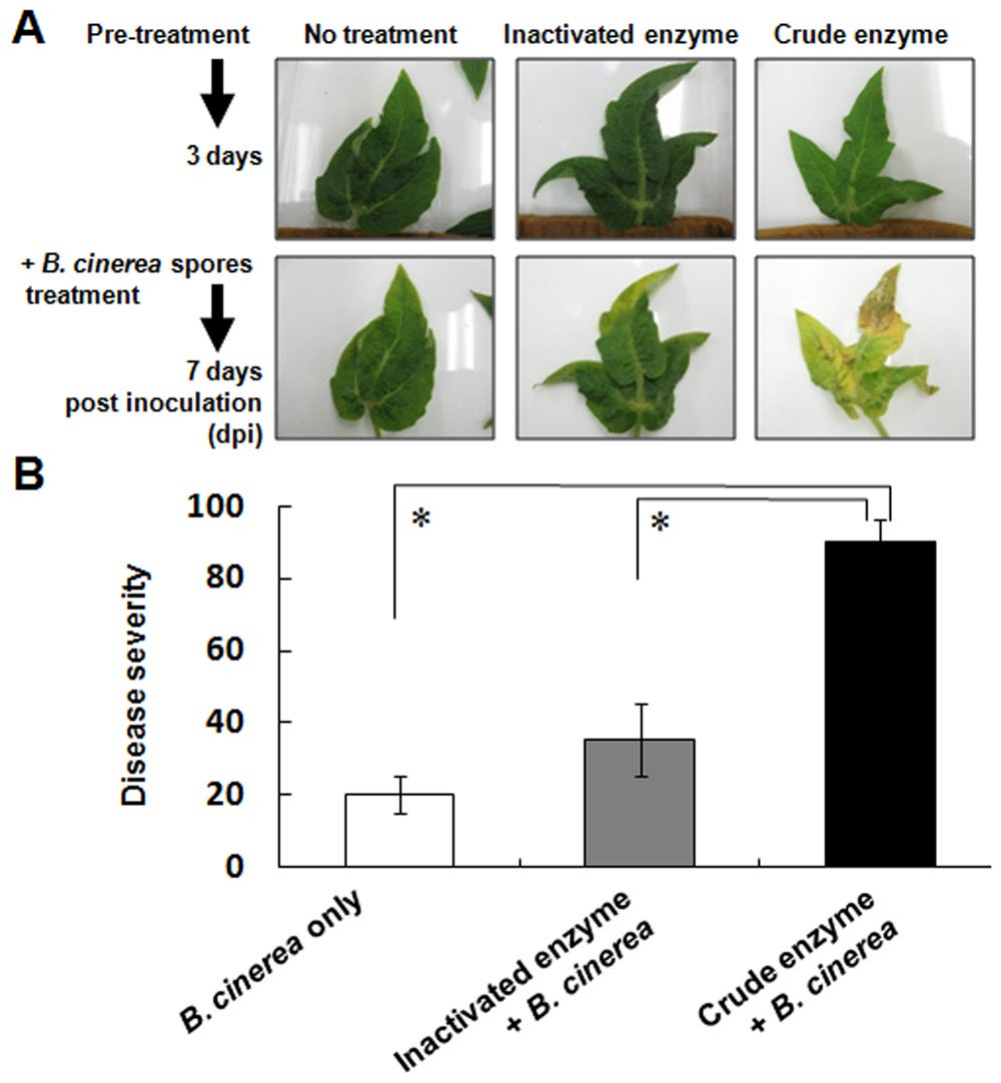
Disease development of *B*. *cinerea* on detached Micro-Tom leaves pretreated with crude or inactivated enzyme solutions. These leaves were inoculated with *B*. *cinerea* spores 3 days after treatment with crude or inactivated enzyme solutions. In the control treatment, only spores were applied. Disease severity on the leaves inoculated with *B*. *cinerea* evaluated at 7 dpi. Data represent mean ± SE of the mean of replicates (n = 4). Asterisks on columns indicate significant differences (*P* < 0.05, one-way ANOVA, Dunnett’s test).

To analyze the effect of crude enzyme treatment on pathogen infectivity on Micro-Tom detached leaves, *B*. *cinerea* spores were inoculated onto Micro-Tom detached leaves at 3 days after crude enzyme treatment. Crude enzyme treatment significantly enhanced chlorotic symptoms in leaves (Fig. 3A) and disease severity [the visual disease symptom scoring system recommended by Japan Plant Protection Association (JPPA)] (Fig. 3B) at 7 dpi (in total, 10 days after crude enzyme treatment) versus those treated with fungal spores alone and those treated with inactivated enzyme. There was no statistically significant difference in disease severity between the leaves treated with inactivated enzyme solution and *B*. *cinerea* compared and those with *B*. *cinerea* alone.

### Spore germination and fungal invasion in crude enzyme-treated Micro-Tom leaves

**Figure 4:**
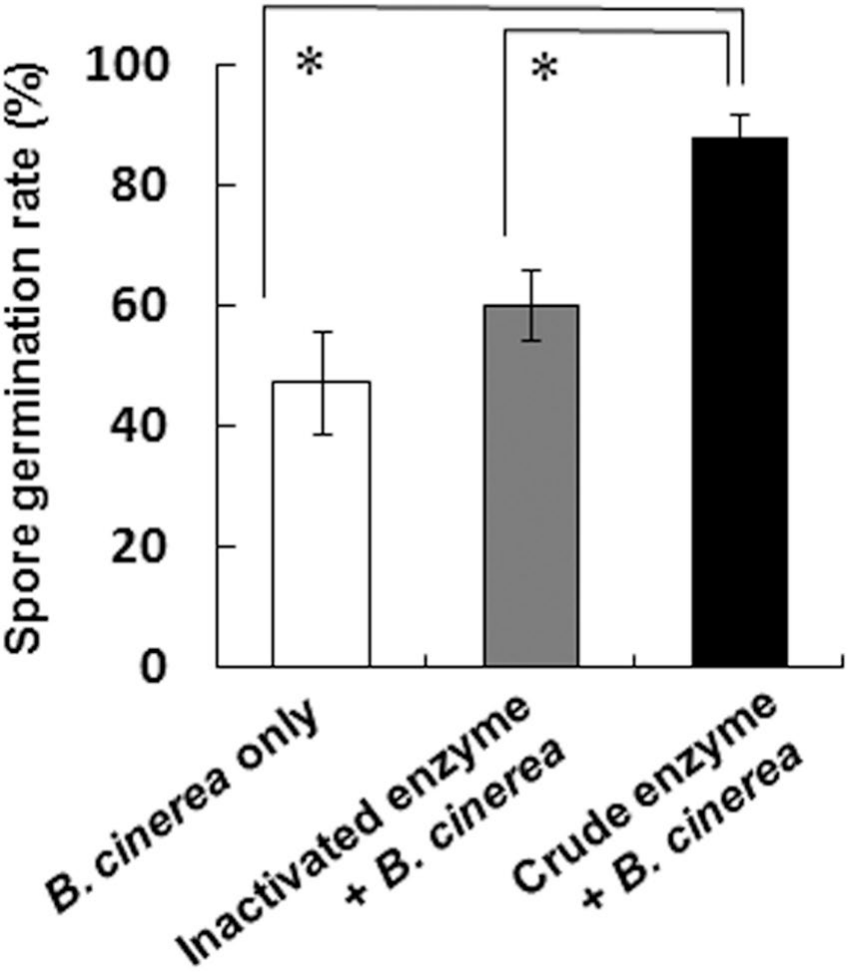
Germination of *B*. *cinerea* spores on Micro-Tom leaves pretreated with crude enzyme solution. The leaves were inoculated with spores at 3 days after each solution treatment and the analysis were done after 2 days. Spore germination rate. Data represent mean ± SE of the mean of replicates (n = 4). Asterisks indicate significant differences (*P* < 0.05, one-way ANOVA, Dunnett’s test).

We observed the development of *B*. *cinerea* spores on detached Micro-Tom leaves under the light microscope at 2 dpi (Fig. 4). *B*. *cinerea* spores inoculated onto crude enzyme pretreated leaves gave a significantly higher germination ratio than that of *B*. *cinerea* only treatment (Fig. 4). Their germ tubes were elongated and their hyphae were spread over the leaves (Figure S3). In contrast, the leaves inoculated with *B*. *cinerea* alone and those treated with inactivated enzyme and fungus treatment showed no difference in the ratio of germinated spores (Fig. 4). Moreover, the germ tubes of most spores in both treatments did not elongate well.

**Figure 5:**
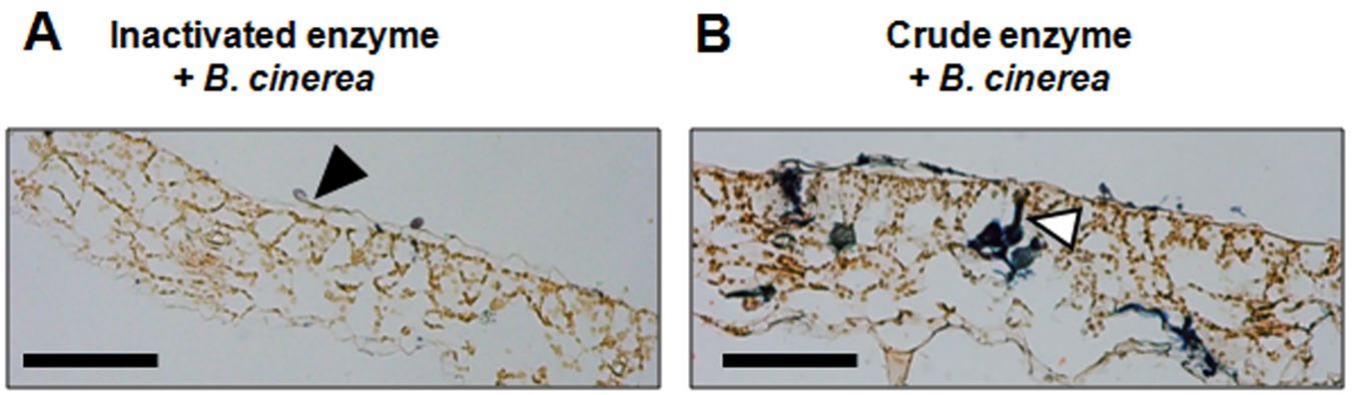
Localization of *B*. *cinerea* cells in cross sectional images of crude enzyme-treated leaves. The spores were inoculated at 3 days after crude enzyme treatment, and the image was taken at 3 days after inoculation. Leaves treated with inactivated (**A**) and active (**B**) crude enzyme solution. Representative image of a germinated spore (closed arrow), and an infected hypha (open arrow). All leaf sections were stained according to Stoughton’s double staining method, staining the fungus blue with thionin and the plant tissue orange with orange G. Scale bar = 1 mm. Similar results were obtained from 4 independent experiments.

**Figure 6:**
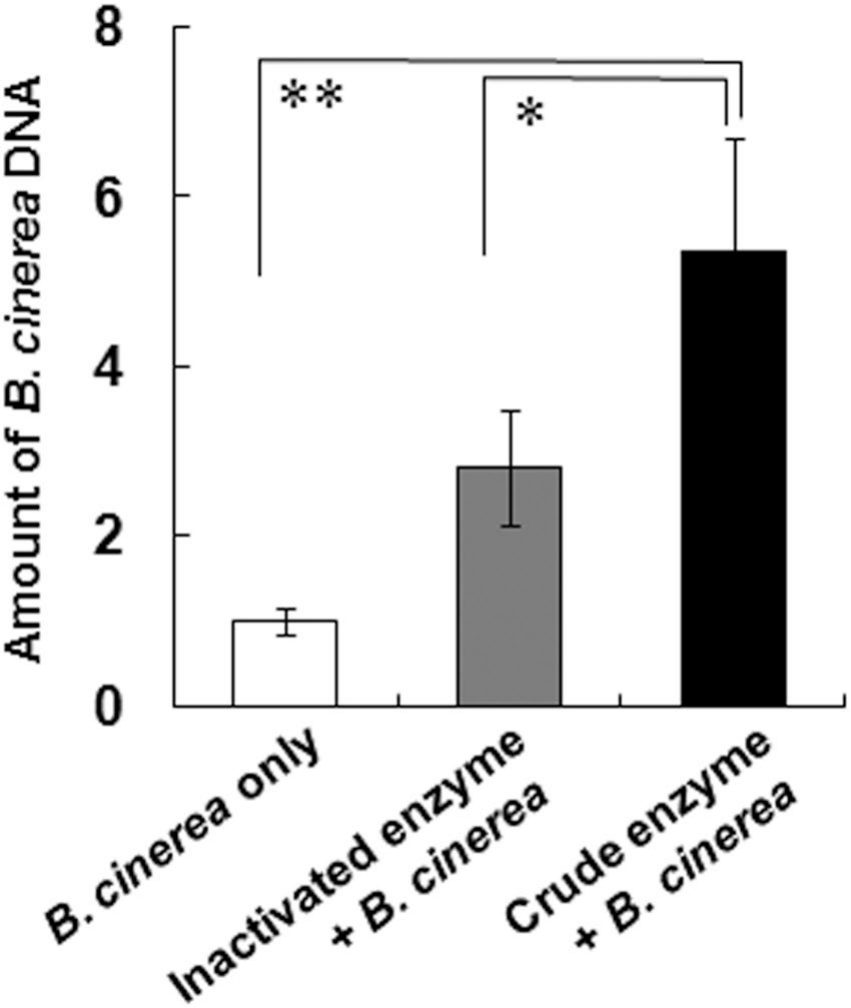
Amounts of *B*. *cinerea* DNA in crude enzyme-treated leaves 3 days after inoculation with *B*. *cinerea* spores. The ratios of *B*. *cinerea* β-tubulin to Micro-Tom *SlACT* were evaluated. All data represent mean ± SE of the mean of replicates n = 5 (spore only) and n = 6 (inactivated or crude enzyme with spores). Asterisks (* and **) indicate significant differences at *P* < 0.05 and *P* < 0.01, respectively (one-way ANOVA, Dunnett’s test).

The penetration of *B*. *cinerea* hyphae into the leaves at 3 dpi was confirmed in a double stained cross-sectional image (Fig. 5) and was quantified fungal DNA (Fig. 6). Vigorously growing hyphae were observed in the tissue on the leaves treated with crude enzyme solution (Fig. 5B). Germinated spores adhered on the leaves treated with inactivated enzyme solution, but the elongated germ tubes did not penetrate into the plant tissue (Fig. 5A). The amount of the fungal DNA on the detached leaves was quantified based on the relative content of *B*. *cinerea* β-tubulin and Micro-Tom actin DNA per given area. The content of fungal DNA was significantly higher after crude enzyme treatment compared with the leaves with no pre-treatment and with inactivated enzyme treatment (Fig. 6).

### Disease severity in crude enzyme-treated Micro-Tom potted plants following inoculation with *B*. *cinerea* spores

**Figure 7:**
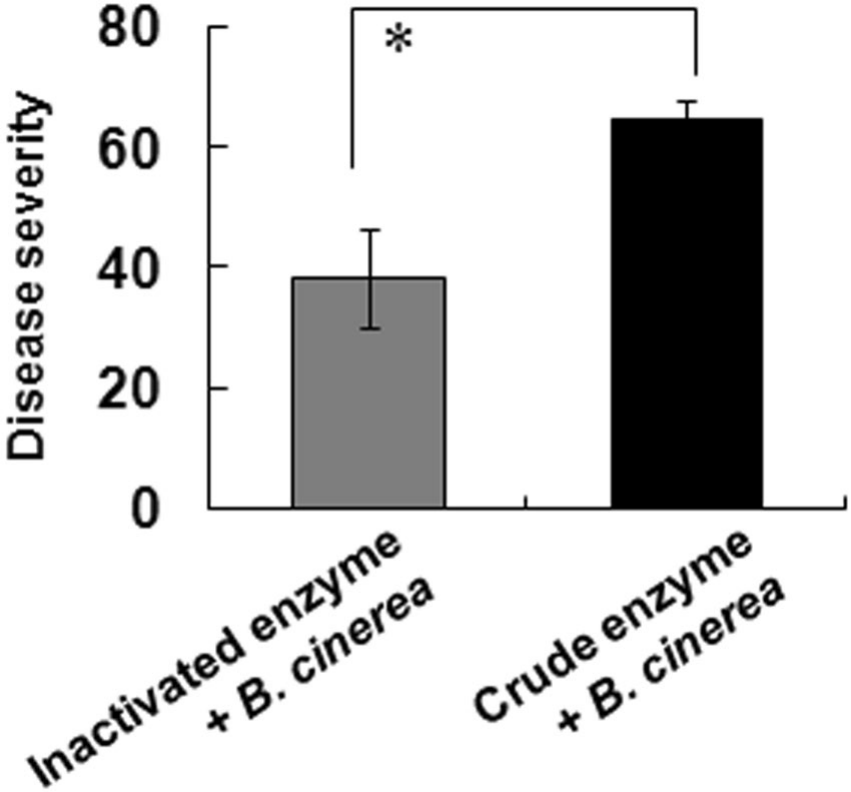
Disease severity in potted Micro-Tom plants treated with crude or inactivated enzyme solutions and *B*. *cinerea* in a glasshouse. *Botorytis cinerea* was inoculated at 3 days after enzyme treatment and disease severity was evaluated at 7 dpi. All data represent mean ± SE of the mean of replicates (n = 4). Asterisk indicates that the data compared were significantly different (*P* < 0.05, Student’s *t*-test).

For analysis at the plant body, we performed a glasshouse scale experiment using Micro-Tom potted plants in the same manner with detached leaves test. Crude enzyme treatment significantly enhanced disease severity at 7 dpi (in total, 10 days after crude enzyme treatment) versus those treated with inactivated enzyme (Fig. 7).

### Effect of crude enzyme treatment on the infectivity of *P*. *polygoni-amphibii* var. *tovariae* on *F*. *japonica* plants

Our results as described above suggested the possibility that crude enzyme treatment could be utilized to enhance infection by *Puccinia* in Japanese knotweed. To assess the feasibility of employing crude enzyme solution as adjuvant for biological control agent, a preliminary experiment was conducted wherein Japanese knotweed was treated with crude enzyme solution prior to inoculation of *Puccinia* spores in a manner, similar to the above investigations. The results were presented in Fig. S4. They showed that in the Japanese knotweed plants pre-treated with crude enzyme solution, some leaves were defoliated even at 0 dpi. Furthermore, the leaves were also damaged similar to our previously reported finding that the crude enzyme treated Micro-Tom potted plants lost most of their leaves and became wilted^10^. Actually, rust symptoms appeared at 3 dpi, which was faster than in plants with no enzyme treatment (control). Crude enzyme treatment significantly promoted disease development at 3 and 6 dpi compared with the control treatment (Figure S4A). The percentage of leaf defoliation increased significantly at 6 and 9 dpi compared with that of plants inoculated with *Puccinia* alone (Figure S4B).

**Figure 8:**
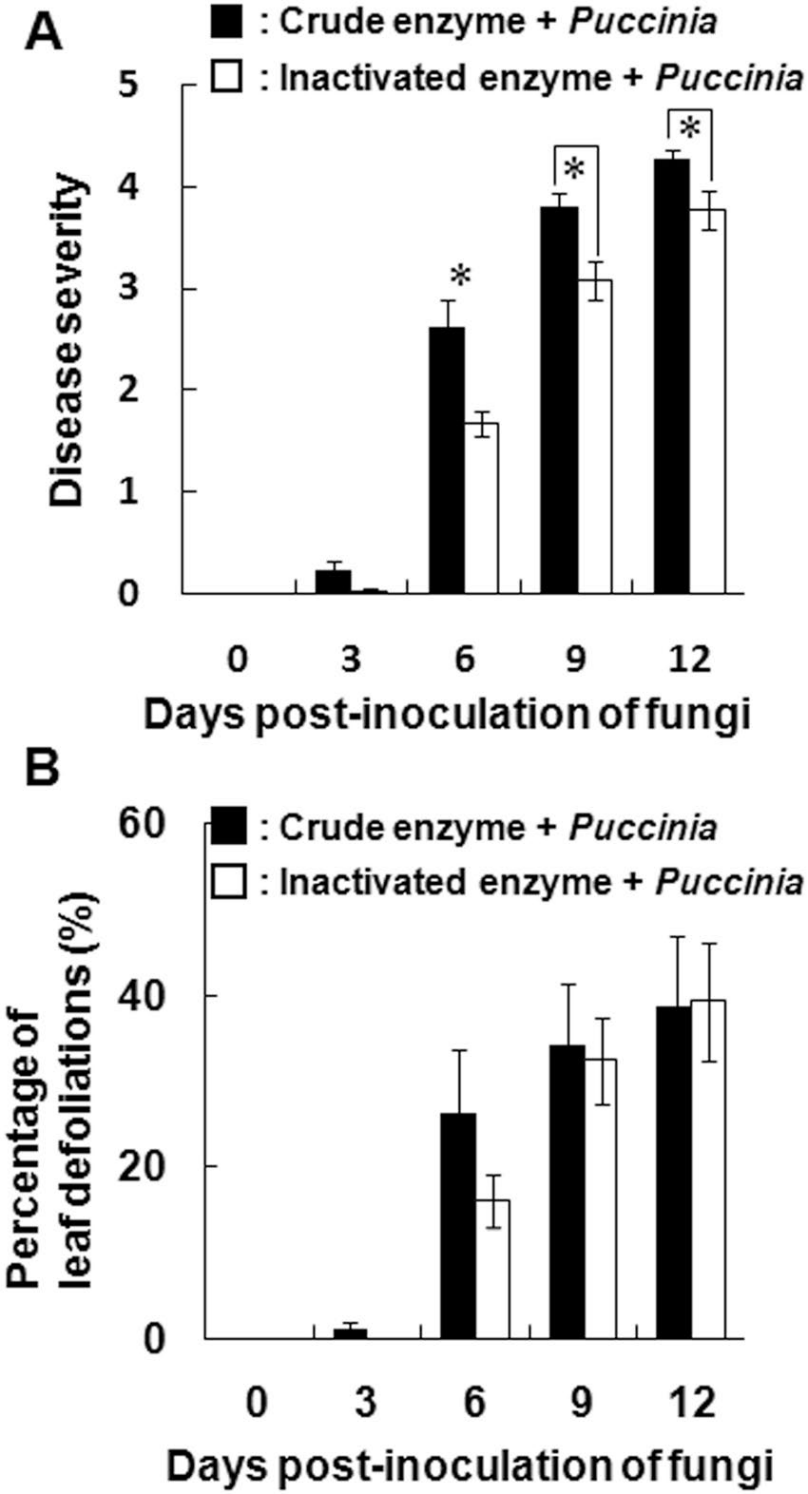
Effects of simultaneous treatment with crude enzyme solution and inoculation of *P*. *polygoni-amphibii* var. *tovariae* on the sensitivity of potted *F*. *japonica*. Rust disease severity (**A**) and percentage of leaf defoliation (**B**) in the test plants. Crude enzyme was applied simultaneously with *P*. *polygoni-amphibii* var. *tovariae*. For the control, inactivated crude enzyme solution was used instead of crude enzyme solution. Asterisks indicate that the values of crude enzyme treatment differed significantly from the control sample (*P* < 0.05, Dunnett’s test). All data represent mean ± SE of the mean of replicates (n = 14).

For the practical application of the crude enzyme for the biological control of Japanese knotweed, the effects of simultaneous treatment with crude enzyme solution and *Puccinia* spores were determined. With coinoculation treatments, application of crude enzyme solution promoted disease development significantly at 6, 9, and 12 dpi compared with the control treatment (inactivated enzyme + *Puccinia*). Severe symptoms were also observed on leaves inoculated with crude enzyme solution with *Puccinia* spores but not on those treated with inactivated enzyme + *Puccinia*. Although disease development at 3 dpi did not differ significantly between treatments of crude enzyme solution with *Puccinia* and the inactivated enzyme + *Puccinia*, more symptoms did appear on the inoculated leaves with the former treatment than on those with the latter (Fig. 8A). Furthermore, defoliated leaves were observed at 3 dpi in the plants coinoculated with crude enzyme solution and *Puccinia*. The percentages of leaf defoliation at 3 and 6 dpi with crude enzyme solution with *Puccinia* were higher than with fungus treatment alone, although the differences were not statistically significant (Fig. 8B).

## Discussion

In this study, we showed that concentrated PaE, an esterase of the phylloplane yeast *P*. *antarctica*, thinned the lipophilic layer of tomato leaves and decreased the leaves’ protection against the plant pathogenic fungus *B*. *cinerea*. Treatment with a PaE-containing culture filtrate (crude enzyme solution) also increased disease development caused by *B*. *cinerea* on tomato plants. We obtained similar results using crude enzyme solution with the rust pathogen *Puccinia* on *F*. *japonica* plants, one of the most serious invasive alien weeds in Europe and North America.

Leaf surfaces are an important habitat for microorganisms but provide limited nutrient resources^3^. On leaf surfaces, these phylloplane yeasts may secrete esterases to extract fatty acids, the major component of the leaf lipophilic layer^12,13^, and assimilate them as carbon source. Based on the affinity of polyclonal antibodies against PaE, we observed that *Pseudozyma* sp. yeasts isolated from vegetable leaves also secrete esterases with high PaE similarity^27^. Previously, we observed that treatment with the crude enzyme thinned the cuticle layer covering the aerial epidermis of leaves^10^. Our crude enzyme solution contained a large amount of PaE and xylanase^11^. Here, we applied purified PaE or a mixture of PaE and xylanase to leaves in the same amounts as contained in crude enzyme solution, and observed that treatments with the solution containing PaE made the cuticle layer thinner, whereas the solution without PaE produced no such effect (Fig. 1). The basidiomycetous yeast *Cryptococcus* species are also often isolated from plant surfaces. *C*. *flavus* and *C*. *magnus* related yeast also secrete about 20 kDa esterases (CfCLE, CmCut1) with 61.0% and 57.3% amino acid sequences identity with PaE, respectively, their BPs degrading activity has been previously reported^6,24^. It indicates that this kind of activity is in general commonly found in cutinase-like enzymes of these yeasts. However, these phylloplane yeasts do not increase disease severity on leaves. In most of leaves, *Pseudozyma* and *Cryptococcus* are found in low populations, which suggests that these phylloplane yeasts secrete small amounts of esterases and utilize small amounts of plant cuticle as nutrients.

The cuticle layer has a role in protecting plants from pathogen invasion as well as acting as a water barrier^28–31^. In an *SlSHIN3*-silenced tomato plant line, loss of function of the transcription factor in regulating cuticle formation and epidermal patterning markedly reduced the thickness of the cuticle layer and increased sensitivity to *B*. *cinerea* infection versus the wild-type plant^32,33^. Similarly, treatment with a solution including PaE reduced the thickness of cuticle layer, and *B*. *cinerea* infection in tomato leaves was enhanced (Figs 2,3 and 7). Because degradation products from the cuticle layer such as cutin monomers induce germination and appressorium formation in phytopathogenic fungi^34^, products released from leaves due to PaE treatment may have triggered the infection structure development of spore (Figs 4–6).

On the leaves treated with inactivated enzyme solution, *B*. *cinerea* infection was enhanced compared to that of *B*. *cinerea* only. The result implies that the crude enzyme treatment compromises the surface barrier of plants against pathogens, leading to enhanced disease development. In addition, we consider that this result could have been due to the induction of fungal growth by residual nutrients derived from *P*. *antarctica* culture medium. It is well known that *P*. *antarctica* has ability to secrete mannosylerythritol lipids that can decrease the contact angle of water droplets and the spreading of microbial cells onto plant surface^35^. It might be possible that these extra components in the PaE active/inactivated crude enzyme solutions also contributed to the enhancement of *B*. *cinerea* infection on the leaves. Furthermore, the results of the experiment where a high concentration of purified *Cryptococcus* CmCut1 was applied on Micro-Tom leaves also enhanced *B*. *cinerea* infection (Figure S5). It is considered that the phenomenon is common for this type of enzymes.

During the plant infection process, *B*. *cinerea* penetrates the cuticle through enzymatic degradation^36^. Because *B*. *cinerea* secretes cutinases, pectin-degrading enzymes, proteases, and laccases on tomato peels^20,37,38^, Suarez *et al*.^36^ and Cotoras and Silva^37^ considered that these enzymes contribute to successful infection. In this study, *B*. *cinerea* easily invaded leaves with damaged cuticles; the enzyme secretion stage of the fungus may not have been required. Our results indicate that the activity of PaE contained in the culture filtrate was responsible for the adjuvant effect of the culture filtrate on fungal infection.

Invasive alien weeds have the potential to spread uncontrolled if no specialized natural enemies are present^39^. Biological control methods have been devised to suppress the propagation of alien weeds. However, biocontrol agent candidates including *Puccinia*, are sometimes not sufficiently damaging to their hosts when applied in the field. We suggested that the method used to support infection in this study would enhance the effectiveness of the biocontrol agents. Based on the observed effect that PaE treatment thinning the plant cuticle layer, we predict that the crude enzyme treatment would improve the weed infectivity of biocontrol agents and boost weed control efficacy. We conducted, therefore, a preliminary investigation to determine the potential of a crude enzyme treatment to control Japanese knotweed (Figure S4). To explore practical, labor-saving applications of this treatment, we examined simultaneous treatment with crude enzyme solution and *Puccinia* spores, and obtained similar results (Fig. 8). These results indicated that the thinning of the physical barrier of the plant surface by crude enzyme treatment enhanced successful infection in target plants before inoculation or during infection. The rapid propagation of the rust fungus on the inoculated area can be expected to lead to significant control of *F*. *japonica* plants in the introduced range. However, further laboratory and also field studies are needed to demonstrate the feasibility of utilizing crude enzyme as an effective adjuvant for biological control. In addition, it would be valuable to test this approach with other weed-biocontrol agent systems.

## Materials and Methods

### Microorganisms

The strain *P*. *antarctica* GB-4(0)-HPM7, stocked in the NARO Genebank as MAFF 30700^10^ was used for production of crude enzyme solution, and preparation of purified xylanase. For preparation of purified PaE, genetically modified strain, *P*. *antarctica* GB-4(0)-X14, that harbors PaE gene (Pa*CLE1*) under the control of strong xylanase promoter (upstream of Pa*XYN1*)^23^ was used.

*Botrytis cinerea* MAFF 305929, that causes gray mold disease of *Solanum lycopersicum*, was obtained from the NARO Genebank (Japan). The strain was grown on potato dextrose agar (PDA) medium (Becton, Dickinson, Sparks, MD) at 18 °C for 2 weeks.

Leaf samples of *F*. *japonica* with disease symptoms of the rust fungus, *P*. *polygoni-amphibii* var. *tovariae*^15^ (*Puccinia*) were collected from a field (Kyushu University, Fukuoka, Japan). The urediniospores mixed with talcum powder (spores:talc = 1:10, w/w) were applied to both leaf surfaces using a fine brush and inoculated plants were kept in a dew chamber with a relative humidity (RH) of 100% at 20 °C without light and incubated for 48 h and then transferred to a glasshouse. After urediniospores matured on the inoculated leaves, they were collected by tapping the leaves and used as the inoculum. The inoculum was kept in the refrigerator before use. In each experiment, the same inoculum was used.

### Plant materials and growth conditions

Plants of *S*. *lycopersicum* (L.) *cv*. ‘Micro-Tom’ (Inplanta Innovations Inc. Ltd., Kanagawa, Japan) were sown on a 1:1 mixture of commercial culture soil (Sakata Super Mix A, Sakata Seed Co. Ltd., Kanagawa, Japan) and vermiculite, and grown under white fluorescent light (2.4 klux) in a 16/8-h light/dark photoperiod at 25 °C at 40–50% RH for 4 weeks.

Rhizomes of Japanese knotweed collected from natural populations growing at Omura, Nagasaki Pref., Japan, were sown in commercial soil (Kenbyo, Yae Nogei Co., Ltd., Nagasaki, Japan) in pots and maintained in a glasshouse at 25 °C under natural light conditions.

### Preparation of crude enzyme solution of the yeast *P*. *antarctica*

The strain *P*. *antarctica* GB-4(0)-HPM7 was cultivated in a jar fermentor with 72-h xylose-fed batch cultivation as described previously^9^. These culture conditions are suitable for *P*. *antarctica* to produce high amount of an esterase (PaE) and xylanase^11^. Crude enzyme solution was prepared by filtration of the culture supernatant using cellulose acetate membrane (pore size 0.45 μm, Toyo Roshi Kaisha, Tokyo, Japan).

### Preparation of purified PaE and xylanase

High concentration of PaE was produced by xylose fed batch cultivation of *P*. *antarctica* GB-4(0)-X14^23^. PaE was purified from the culture filtrate without concentration by the affinity chromatography using poly(butylene succinate-*co*-adipate) (PBSA) (Bionolle EM-301, average molecular weight 12–15 × 10^4^; Showa Denko K. K., Tokyo, Japan) as carrier, which was developed previously^24^. The purification of xylanase was performed from culture filtrate of *P*. *antarctica* GB-4(0)-HPM7 (crude enzyme solution) as follows: the crude enzyme solution was passed through a Mono Q HR 5/5 column (GE Healthcare Life Science, Little Chalfont, UK), and the flow-through fraction was concentrated 24 times by ultrafiltration. The filtrate (100 μl) was mixed with 900 μl of 50 mM Na-phosphate buffer (pH 7.1) containing 1.3 M ammonium sulfate. The mixture (1 ml) was applied to a Phenyl Superose HR 5/5 column (GE Healthcare Life Science). The xylanase was isolated in a fraction of a linear gradient (1.2–0 M) of ammonium sulfate in 50 mM sodium phosphate buffer (pH 7.1) as described previously^11^.

### Enzyme activity

Since PaE was first isolated and identified as BPs degrading enzymes, PaE activity was evaluated based on the decrease in turbidity of emulsified PBSA as described previously^27^. One unit of the PBSA-degrading activity was defined as a 1 OD_660_ decrease per min in 20 mM Tris-HCl buffer (pH 6.8) at 30 °C.

The xylanase activity was determined using xylan from birch wood as the substrate, and after the reaction, the amount of reducing sugar released was analyzed by modified Somogyi–Nelson method^11,40,41^. One unit of xylanase activity was defined as 1 µmol of _D_-xylose liberated per min in the 15 mM phosphate buffer (pH 7.1) at 30 °C.

### Treatment of tomato plants and leaves with enzyme solution

PaE shows a relatively high PBSA degradation activity under alkaline conditions.

The crude enzyme solution was adjusted to 8 U of PaE activity by dilution with 20 mM Tris-HCl buffer (pH 8.8) just before use (generally, the final buffer concentration was 2.22 mM). Under this condition, the crude enzyme solution had 58 U of xylanase activity. Purified PaE and xylanase were adjusted to 8 U and 58 U solutions, respectively, using 20 mM Tris-HCl buffer (pH 8.8). Under at pH 8.8, the activity of PaE has about 4 times of the pH 6.8, and the activity of xylanase has about 70% of the one at pH 7.1. Mixture of PaE and xylanase was also prepared in the same buffer. The crude enzyme solution was heated at 121 °C for 15 min, and after confirming lost the PaE activity, it was used as the inactivated enzyme solution. Inactivated enzyme solution, diluted buffer and SDW were used as control of each experiment.

A droplet (10 µl) of adjusted purified enzyme solution was spotted on the left side of each detached Micro-Tom leaf and a droplet of diluted buffer on the opposite side of the leaf as a control. The detached leaves were then kept in closed containers under high humidity conditions at 25 °C for 3 days.

About 600 µl of crude enzyme solution or inactivated enzyme solution per leaf was sprayed over the upper side using pump sprayer. The potted Micro-Tom plants which resulting in a total of 10 ml of solution sprayed were incubated in a glasshouse at an average temperature of 25 °C at 40–50% RH for 3days. The detached Micro-Tom leaves were kept at 25 °C under high humidity conditions in closed containers for 3 days.

The incubated samples were subsequently inoculated with *B*. *cinerea* spores.

### Thickness measurement of lipophilic layer of enzyme-treated leaves

Three days after treatment of the purified enzyme solution, leaves were cut into 1 cm × 1 cm fragments. The piece of leaf was held between slit of pith and sectioned by razor manually. The lipophilic layer of the leaf was stained with Sudan III (Sigma-Aldrich) according to Buda *et al*.^42^ with some modifications as follows: Leaf sections were soaked in the filtered Sudan III solution (2% w/v in 70% ethanol) for 8 min and were then rinsed first with 50% ethanol, followed by distilled water twice. Stained sections were mounted and observed under an ECLIPSE Ni light microscope with DS-Fi2 CCD camera system, photographed, and thickness of stained lipophilic layer was measured by NIS Elements ver. 4.40 software (Nikon, Tokyo, Japan).

### Inoculation of *B*. *cinerea* spores onto tomato plants and detached leaves

Spores of *B*. *cinerea* appearing on PDA culture plates were suspended in SDW at a final concentration of 5 × 10^4^ spores ml^−1^. On the crude enzyme- or inactivated enzyme-treated leaves, prepared as described above, ~600 µl of the spore suspension was sprayed over the upper side of each leaf using a pump sprayer (theoretically, 3 × 10^4^ spores inoculated onto the surface of a leaf). Then, the potted plants and detached leaves were kept under the conditions described above. For the control treatment, spores were inoculated onto plants without pretreatment with crude or inactivated enzyme solutions.

### Observation of *B*. *cinerea* spore germination on leaf surfaces

After inoculation of *B*. *cinerea* spores, the detached leaves were incubated for 3dpi at 25 °C under high humidity conditions. Then, a spore suspension was sprayed on the surface of the detached leaves, and incubated for 2 dpi. The leaf was cut into 1 × 1-cm squares and the cut leaves were boiled for 1 min in a lactophenol/cotton-blue solution (Sigma-Aldrich). To remove excess stain, the cut-leaves were submerged in 2.5 g ml^−1^ chloral hydrate (Wako, Osaka, Japan) overnight^43^. In each treatment, more than 100 stained spores were observed per cut leaf under the light microscope. We calculated the ratio of germinated spore to total number of spore. Four cut leaves were used in each trial.

### Distribution of *B*. *cinerea* cells in leaf sections treated with crude enzyme solution and *B*. *cinerea* spores

Detached leaves treated with crude enzyme solution and *B*. *cinerea* spores were incubated for 3 dpi under the same conditions. Each leaf was fixed with FAA solution (5% formaldehyde, 70% ethanol, and 5% acetic acid) for 48 h. The fixed samples were dehydrated with ethanol (70% for 24 h and 85% for 3 h) and subsequently displaced with butanol through a butanol solution series (35% for 1 h, 55% for 1 h, 75% for 1 h, and 100% for 24 h). Samples were then embedded in Paraplast Plus (Sigma-Aldrich, St. Louis, MO). Tissue sections (15 μm thick) were prepared using a rotary microtome (Leitz, Austria). The samples were dewaxed in xylene, and treated sequentially with anhydrous ethanol and 70% ethanol. Then, the samples were stained according to Stoughton^44^ with some modifications. First, staining was done with 0.025% (w/v) thionin acetate (Sigma-Aldrich) in 0.1 mM sodium acetate and a saturated solution of Orange G dye (Sigma-Aldrich), before rinsing with a solution of ethanol and xylene (1:1). Stained sections were mounted on glass slides and observed under a BX50 light microscope with an FX380 CCD camera system (Olympus, Tokyo, Japan).

### Quantification of *B*. *cinerea* DNA in crude enzyme-treated leaves

Detached leaves inoculated with *B*. *cinerea* spores were incubated for 3 dpi under the same conditions. Then, about 100 mg of leaves were frozen in liquid nitrogen and each leaf sample was ground separately using a mortar and pestle. From the resulting powder, DNA from each sample was extracted with cetyltrimethyl ammonium bromide (CTAB) method^36^ as follows. Ground samples were mixed with 600 µl of homogenizing buffer (2% CTAB, 50 mM Tris-HCl (pH 8.0), 20 mM EDTA, 1.4 M NaCl, and 1% PVP). After incubation at 65 °C for 15 min, chloroform/isoamyl alcohol (24:1) extractions were performed twice. Nucleic acid was precipitated from the aqueous layer by the addition of 0.7 volumes of isopropanol. After centrifugation, the pellet was washed with 70% (v/v) ethanol, vacuum-dried, and redissolved in 100 µl of TE buffer with 0.25 units of RNase H (Takara Bio, Shiga, Japan).

*Botorytis cinerea* β-tubulin DNA (accession no. KC620303) in the extracted genomic DNA was quantified by real-time PCR using specific primers [forward, 5′-GTTACTTGACATGCTCTGCCATT-3′; reverse, 5′-CACGGCTACAGAAAGTTAGTTTCTACAA-3′]^45^. Similarly, the DNA for Micro-Tom actin (*SlACT*, accession no. FJ532351) was quantified with specific primers [forward, 5′-CCAGGTATTGCTGATAGAATGAG-3′; reverse, 5′-GAGCCTCCAATCCAGACAC-3′]^7^. The PCR was carried out with 100 ng of total DNA using the GeneAce SYBR qPCR Mix α No ROX (Nippongene, Toyama, Japan) on a RealTime-PCR System LightCycler Nano (Roche, Basel, Switzerland) as recommended by the supplier. The thermal cycling program was as follows: 95 °C for 10 min, then 45 cycles of 95 °C for 15 s and 60 °C for 30 s. The total DNA content of each leaf was normalized to tomato *SlACT* content, and the ratio of fungal gene content to total DNA was calculated. Seven leaves were analyzed for each treatment.

### Treatment of *F*. *japonica* plants with crude enzyme solution and *P*. *polygoni-amphibii* var. *tovariae*

In this experiment, crude enzyme solution and spore suspension was used simultaneously. For coinoculation treatments, spore suspensions containing 1.5 × 10^5^ spores ml^−1^ in crude enzyme solution that was adjusted as described above were sprayed on both the adaxial and abaxial sides of leaf surfaces. Control plants were inoculated with spores in inactivated enzyme solution.

After the treatments, all the inoculated plants, together with the control plants, were transferred to a dew chamber (KI Holdings Co., Ltd., Yokohama, Japan) with an RH of 100% at 20 °C without light and incubated for 48 h. After removal of the plants from the chamber, they were maintained in the glasshouse.

Seven plants with 2–10 leaves were used for each treatment, the experiments were repeated twice. After treatment with crude enzyme solution, observations of disease severity and percent leaf defoliation were assessed every 3 days until 12 dpi in both experiments.

### Evaluation of disease severity

Tomato plants: After inoculation of *B*. *cinerea* spores, the potted plants were incubated for 7 days post-inoculation (dpi) in a glasshouse. Then, disease severity was evaluated according to the following the recommendations of the Japan Plant Protection Association (JPPA) (http://www.jppa.or.jp/index.html) and the following five-step index: 0, no visible lesion; 1, one or two lesions; 2, <25% of leaf area lesioned; 3, 25–50% of leaf area lesioned; or 4, >50% of leaf area lesioned. Disease severity per plant was then calculated using the following formula: Σ (number of diseased leaves per rating × rating value) × 100/(4 × total number of leaves).

*F*. *japonica* plants: The pathogenic activity of the *Puccinia* obtained from the stocked leaves was evaluated on *F*. *japonica* in glasshouse experiments based on synergism significance as the rust disease symptom. Disease symptoms of the rust fungus were evaluated based on a disease severity index, according to the following scale: (0) no symptoms; (1) yellow spot without forming uredinia; (2) 1–50% of diseased area with formation of uredinia per leaf; (3) >50% of diseased area with formation of uredinia per leaf; (4) leaf chlorosis; or (5) leaf defoliation. Disease severity per plant was then calculated using the following formula: Σ (number of diseased leaves per rating × rating value)/total number of leaves per plant. The percentage of leaf defoliation was counted as the ratio of the number of defoliated leaves to the total number of leaves per plant. Mean disease severity and mean percentage of leaf defoliation were averaged over the total number of plants.

### Statistical analyses

For the study of tomato plants, the disease severity data were analyzed using Student’s *t*-test. The spore germination rate was arcsine-transformed before performing analysis of variance (ANOVA). The resulting data were analyzed by ANOVA before using Dunnett’s test to compare means. The quantification of fungus DNA data was analyzed by ANOVA before using Dunnett’s test to compare means. These data were analyzed using ‘R’ software (ver. 3.2.4; The R Foundation for Statistical Computing, Vienna, Austria).

For experiments using *F*. *japonica*, the statistical analyses were performed using JMP software (ver. 7 for Windows; SAS Institute Inc., Cary, NC, USA). The percentage of leaf defoliation was arcsine-transformed before performing ANOVA. The resulting data from each experiment were analyzed by ANOVA before using Dunnett’s test to compare means.

### Compliance with Ethical Standards

This article does not contain any studies with human or animal subjects performed by any of the authors.

## Electronic supplementary material

Supplementary information
