# Supplementary material for: Disease severity enhancement by an esterase from non-phytopathogenic yeast Pseudozyma antarctica and its potential as adjuvant for biocontrol agents
Source: Sci Rep. 2018 Nov 7;8:16455. doi: 10.1038/s41598-018-34705-z (PMC6220330; doi:10.1038/s41598-018-34705-z)
Supplement: Supplementary file 1 — Supplementary information [file 41598_2018_34705_MOESM1_ESM.pdf]

Supplementary Material

Disease severity enhancement by an esterase from non-phytopathogenic yeast *Pseudozyma antarctica* and its potential as adjuvant for biocontrol agents

Hirokazu Ueda[1], Daisuke Kurose[1§], Soichi Kugimiya[1], Ichiro Mitsuhara[1], Shigenobu Yoshida[1], Jun Tabata[1], Ken Suzuki[1], and Hiroko Kitamoto[1*]

1 National Agriculture and Food Research Organization (NARO), Kan-nondai, Tsukuba, Ibaraki, Japan

Correspondence; NARO, 3-1-3 Kan-nondai, Tsukuba, Ibaraki 305-8604, Japan. E-mail: kitamoto@affrc.go.jp.

[§]Present address; CABI Europe-UK, Bakeham Lane, Egham, Surrey TW20 9TY, UK

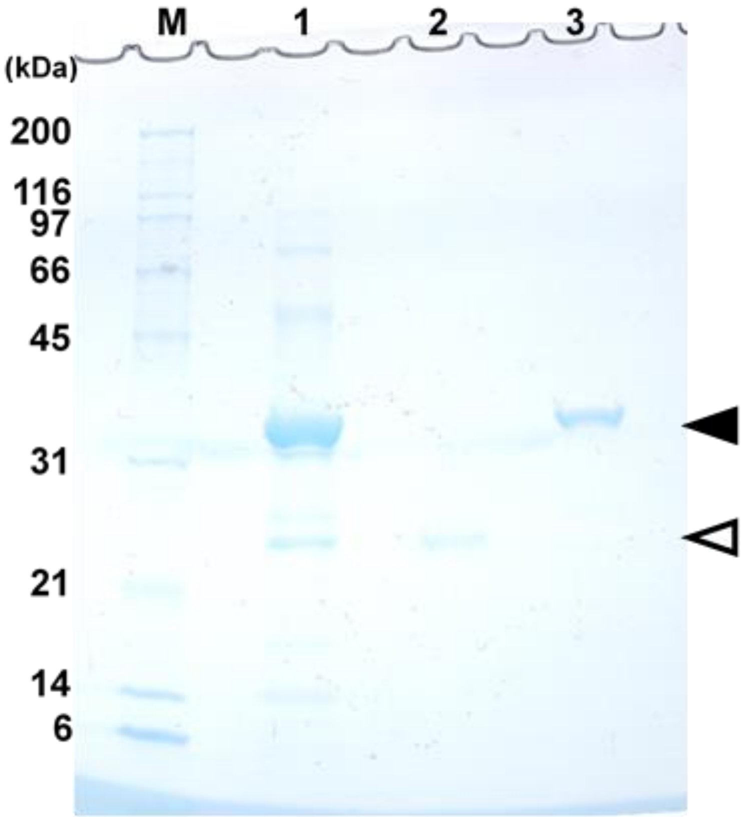

Figure S1. SDS-PAGE of the purified PaE, xylanase and the culture filtrate of *P. antarctica*. *Lane M*: molecular weight protein markers; *lane 1*: culture filtrate (crude enzyme solution) (5 µl); *lane 2*: purified PaE solution (0.05 µl); *lane 3*: purified xylanase solution (0.1 µl). The open arrow and closed arrow indicates the position of PaE and xylanase, respectively.

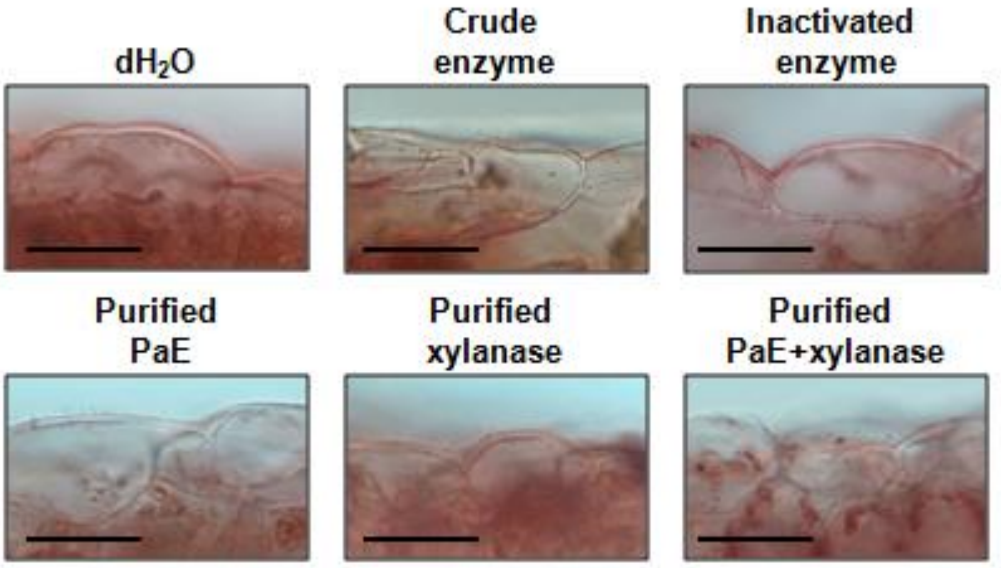

Figure S2. Cross sectional images of Sudan III-stained cuticle layer of Micro-Tom leaves treated with dH_2O, crude enzyme solution, inactivated crude enzyme solution, purified PaE, purified xylanase and mixture of purified PaE and xylanase. Scale bar= 500 μm. Similar results were obtained from 4 independent experiments.

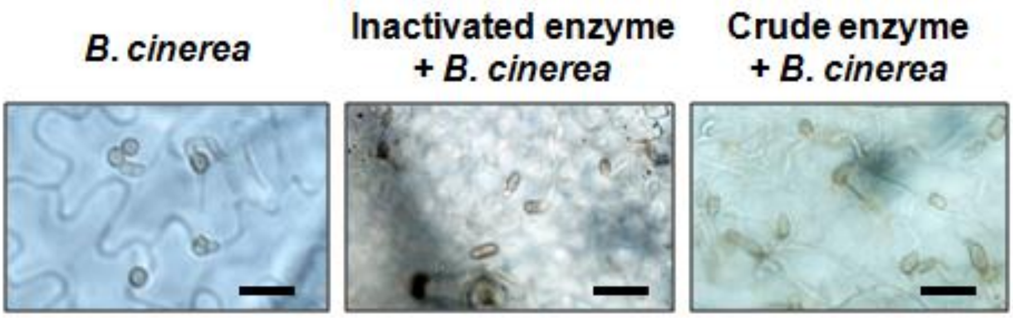

Figure S3. Morphology of germinated *B. cinerea* spores on Micro-Tom leaves pretreated with crude enzyme solution. The leaves were inoculated with spores at 3 days after crude enzyme treatment and the analysis were done after 2days. Scale bar = 100 μm.

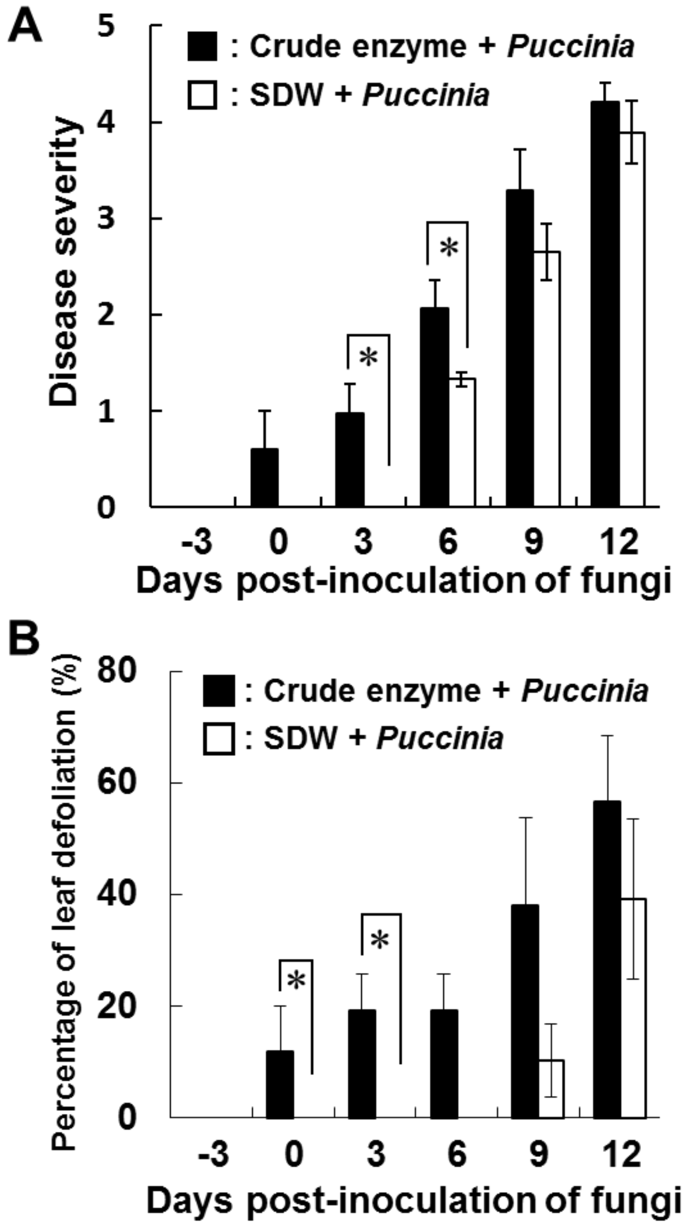

Figure S4. Effect of crude enzyme pre-treatment on sensitivity of potted *F. japonica* plants to infection by *P. polygoni-amphibii* var. *tovariae*. Rust disease severity (**A**) and percentage of leaf defoliation (**B**) in the test plants. Crude enzyme solution was applied 3 days before inoculation of *P. polygoni-amphibii* var. *tovariae*. For the control, sterilized distilled water was used instead of the crude enzyme solution. Asterisks indicate that the values of crude enzyme treatment were significantly different from the control sample ($P < 0.05$, Dunnett's test). All data represent mean ± SE of the mean of repeated experiments (n = 5 of SDW with spores and 6 of crude enzyme with spores).

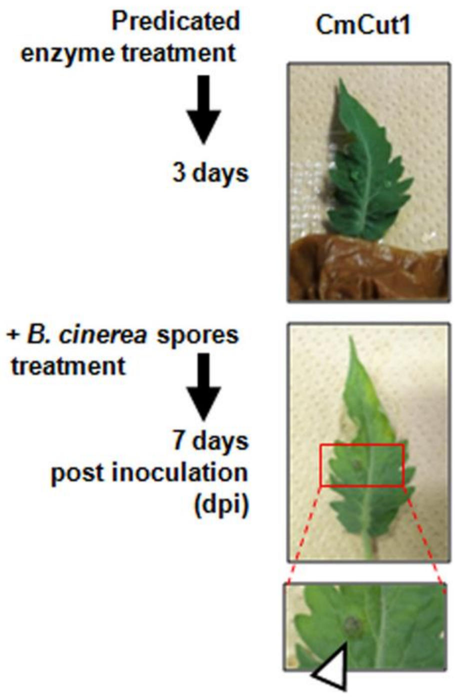

Figure S5. Disease development of *B. cinerea* on detached Micro-Tom leaves following treatment with the purified CmCut1 solution. The CmCut1 solution and buffer were spotted on the left and right sides of the leaves, respectively. After 3 days, the *B. cinerea* spores were inoculated. Representative images of leaves 7 days after *B. cinerea* inoculation. Bottom: enlarged images of the enzyme-treated area. Open arrow indicates a disease symptom. Data were reproducible (n = 3).
